# Sensors Based on Molecularly Imprinted Polymers in the Field of Cancer Biomarker Detection: A Review

**DOI:** 10.3390/nano14161361

**Published:** 2024-08-19

**Authors:** Camila Quezada, S. Shiva Samhitha, Alexis Salas, Adrián Ges, Luis F. Barraza, María Carmen Blanco-López, Francisco Solís-Pomar, Eduardo Pérez-Tijerina, Carlos Medina, Manuel Meléndrez

**Affiliations:** 1 Department of Materials Engineering (DIMAT), Faculty of Engineering, Universidad de Concepción, Edmundo Larenas 315, Box 160-C, Concepción 4070409, Chile; ssaireddy@udec.cl (S.S.S.); ages@udec.cl (A.G.); 2 Department of Mechanical Engineering (DIM), Faculty of Engineering, University of Concepción, 219 Edmundo Larenas, Concepción 4070409, Chile; alesalas@udec.cl (A.S.); cmedinam@udec.cl (C.M.); 3 Department of Biological and Chemical Sciences, Faculty of Medicine and Science, Universidad San Sebastián, General Lagos 1163, Valdivia 5090000, Chile; luis.barraza@uss.cl; 4 Department of Physical and Analytical Chemistry, Asturias Biotechnology Institute, University of Oviedo, 33006 Oviedo, Spain; cblanco@uniovi.es; 5 Centro de Investigación en Ciencias Físico Matemáticas, Facultad de Ciencias Físico Matemáticas, Universidad Autónoma de Nuevo León, Av. Universidad s/n, San Nicolás de Los Garza 66455, Mexico; francisco.solispm@uanl.edu.mx (F.S.-P.); eduardo.pereztj@gmail.com (E.P.-T.); 6 Facultad de Ciencias para el Cuidado de la Salud, Universidad San Sebastián, Campus Las Tres Pascualas, Lientur 1457, Concepción 4060000, Chile

**Keywords:** detection of tumor markers, molecularly imprinted sensors, molecularly imprinted polymers, cancer biomarkers

## Abstract

Biomarkers play a pivotal role in the screening, diagnosis, prevention, and post-treatment follow-up of various malignant tumors. In certain instances, identifying these markers necessitates prior treatment due to the complex nature of the tumor microenvironment. Consequently, advancing techniques that exhibit selectivity, specificity, and enable streamlined analysis hold significant importance. Molecularly imprinted polymers (MIPs) are considered synthetic antibodies because they possess the property of molecular recognition with high selectivity and sensitivity. In recent years, there has been a notable surge in the investigation of these materials, primarily driven by their remarkable adaptability in terms of tailoring them for specific target molecules and integrating them into diverse analytical technologies. This review presents a comprehensive analysis of molecular imprinting techniques, highlighting their application in developing sensors and analytical methods for cancer detection, diagnosis, and monitoring. Therefore, MIPs offer great potential in oncology and show promise for improving the accuracy of cancer screening and diagnosis procedures.

## 1. Introduction

Cancer is a group of extremely prevalent diseases characterized by the uncontrolled proliferation of cells and the subsequent spread of these cells to healthy tissues. Annually, the global incidence of cancer surpasses 11 million individuals, with 4 million additional cases detected in 2020 [1]. According to a report published by the World Health Organization (WHO), the total number of fatalities in 2020 was estimated to be around 10 million [2]. In 2022, it is anticipated that there will be 1,918,030 new cases of cancer and 609,360 cancer-related deaths in the United States, including approximately 350 deaths per day from lung cancer, the leading cause of cancer-related death [3]. There are about 200 unique forms of cancer, including prostate (more common in men), breast (more common in women), lung, skin, ovarian, hematologic, gastric, colon, and leukemia. Environmental (tobacco, radiation, alcohol, chemicals) and genetic (inherited and autoimmune mutations) factors, as well as bacterial or viral infections, may be responsible for cancers. This uncontrolled cell growth leads to the production of tumor cells that are resistant to apoptosis and other growth regulatory processes in the body, resulting in the formation of tumors that ultimately transcend the body’s homeostatic checkpoints. Once the tumor has spread and begun to metastasize, the cancer has essentially reached an incurable stage and can only be slowed or treated with intensive medicine and careful supervision [4].

Physical examination for lumps, imaging techniques such as X-rays, magnetic resonance imaging (MRI), computed tomography (CT), ultrasound, positron emission tomography (PET), and biopsy are current standard diagnostic techniques [5]. To address these problems, there is an increasing trend toward the usage of molecular techniques including genomic and proteomic materials, enzyme-linked immunosorbent assay (ELISA), polymerase chain reaction (PCR), immunohistochemistry (IHC), radioimmunoassay (RIA), and flow cytometry analysis [6,7,8]. Despite the high sensitivity and accuracy exhibited by these specialized biomarker-based methodologies, their implementation is hindered by their costly and labor-intensive nature. Furthermore, it is worth noting that these treatments frequently fail to yield timely results, posing challenges for early detection of cancer. Hence, there is an urgent need to fabricate, develop, and discover novel diagnostic tools. The development of such devices is vital in addressing the existing challenges of traditional clinical diagnostics, which often involve complex and expensive equipment.

Cancer cells exhibit a diverse range of genetic changes, such as gene rearrangements, point mutations, and gene amplifications. These changes disrupt the molecular pathways that regulate cell growth, survival, and metastasis. The occurrence of such alterations in most patients with a certain type of tumor allows these changes to be used as biomarkers to detect and build targeted therapies, as well as to predict how different treatments will affect patients [9]. The presence or absence of pathogen infection in biological processes can be determined by biomarkers, which function similarly to indicators. Therefore, biomarkers play an important role in the diagnosis and prognosis of cancers. Biomarkers can be found in blood, urine, sweat, and tears. To reliably identify cancer biomarkers, innovative bio-affinity sensors have been continuously investigated and effectively employed as an alternative option to overcome most of the complications described above and have become useful analytical tools. Numerous biosensors have been developed for the purpose of detecting cancer-associated biomarkers. These biosensors primarily employ biorecognition elements, including antibodies, aptamers, enzymes, peptides, DNA, RNA, etc. However, an emerging alternative to these natural biorecognition elements is the utilization of molecularly imprinted polymers (MIPs), which serve as synthetic antibodies in biosensing applications [10,11], simulating the lock and key principle present in biological systems [12].

This review aims to provide an overview of the potential applications of these biomarkers in advancing sensors employing molecularly imprinted polymers (MIPs) that have been used for the identification of cancer biomarkers over the last decade. Subsequently, recent advances, limitations, and prospects are discussed.

## 2. Molecularly Imprinted Polymers

**Figure 1:**
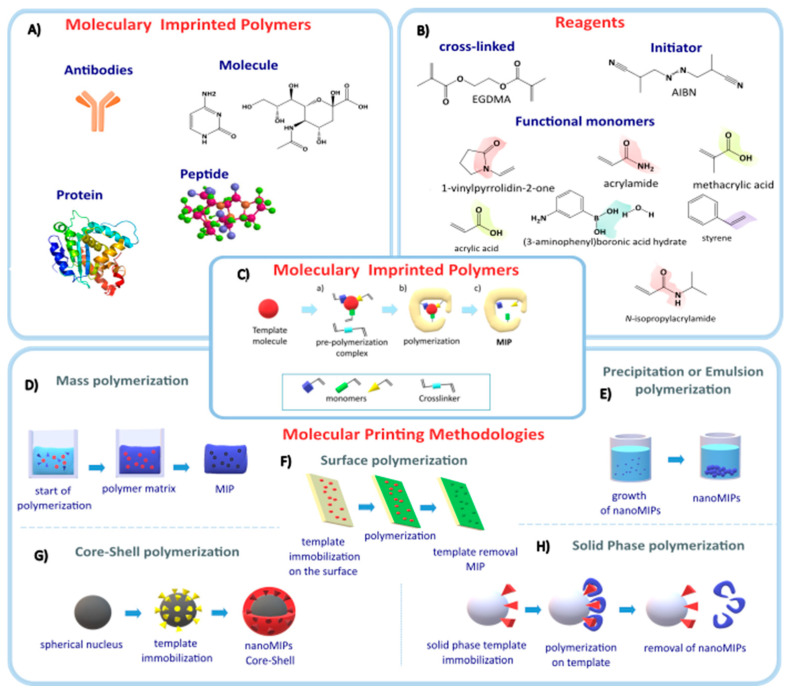
(**A**) Template molecules. (**B**) Reagents used in the molecular printing process. (**C**) General scheme of molecular imprinting polymers, from (**a**–**c**), the pre-polymerization, polymerization, and template extraction complex, is presented. (**D**–**H**) Scheme of the most common molecular printing methods.

Molecularly imprinted polymers (MIPs) are artificial receptors made of polymers synthesized in the presence of a target molecule, mainly known as a template molecule (Figure 1A). MIPs are created by polymerizing functional monomers and cross-linkers (Figure 1B) in the presence of a template molecule. When the template is removed, cavities are formed that are structurally and electrostatically complementary to the template molecule [13]. MIPs function similarly to natural antigen and antibody systems, using a “lock and key” mechanism to bind to the molecule with which they were selectively synthesized. MIPs exhibit the same level of specificity and selectivity as biological receptors, with additional benefits such as cost-effectiveness and sustainability. The synthesis techniques for MIPs are shown in Figure 1C and can be summarized as follows:

(A) The preparation of MIPs requires the interaction of a template (target molecule) that interacts with the functional groups of the monomer and a cross-linking agent.
(B) Polymerization is initiated, and then the template molecule is removed from the acquired polymer, leaving behind the imprinted cavities to bind to the specific target.
(C) The presence of the target analyte in the sample leads to selective absorption by MIPs [14,15,16].

Molecularly imprinted polymers (MIPs) have been extensively investigated for various purposes by researchers in biomedical fields, such as the analysis of different natural compounds [17,18,19], drug molecules [20,21], amino acids [22,23], proteins [15,16], bacteria [24], and viruses [25]. Due to the enormous advantages offered by MIPs, much attention has been paid to developing MIPs to detect and treat various types of cancer [26,27,28]. Using biomarker templates in MIPs has demonstrated considerable efficacy as a cancer detection technique. MIPs have demonstrated their applicability in cancer research and timely disease detection, leading to a better understanding of their underlying mechanisms. The following section describes the different methods of MIP synthesis.

### 2.1. MIP Synthesis Methods

#### 2.1.1. Bulk Polymerization

Various techniques exist for the synthesis of MIPs, with the choice of method dependent upon the specific nature of the target molecules and the intended applications of the resulting MIPs. Additionally, the synthesis yield highly depends on the monomers, cross-linkers used, and the synthesis methodology. One of the first synthesis methods was bulk polymerization. In this synthetic process, the template is mixed with the monomers and the initiator, generating a highly viscous polymeric matrix, leaving the template randomly distributed throughout the polymer (Figure 1D). Finally, a bulk polymer is produced that must be crushed and sieved, resulting in MIPs of different shapes and sizes ranging from millimeters to microns. One potential drawback of employing this methodology is the loss of binding sites during the grinding process. Consequently, it may not always be feasible to completely extract the template from the polymer due to its retention within solvent-inaccessible sites.

Another method of synthesis involves a suspension-based polymeric technique. In this process, the monomers and initiator are dissolved in an organic medium, while the template is dissolved in an aqueous medium. This results in the formation of two distinct phases within the reaction mixture. Through the application of vigorous and constant stirring, the MIPs initiate their formation in the form of small droplets at the interface between the two phases [29,30,31,32]. If the objective is to acquire spherical particles, alternative techniques may prove more advantageous due to their enhanced regulation of chain growth [33,34], such as precipitation, emulsion, core-shell, surface printing, or solid-phase methods [7,8,9,10], which will be described below.

#### 2.1.2. Precipitation Polymerization

In precipitation polymerization, an excess of solvent is employed, resulting in a higher dilution of the reactants. Consequently, the polymer chains being formed do not fully occupy the solvent volume, forming a dispersion of spherical particles (Figure 1E) [35,36,37]. This polymerization technique has been widely employed in the synthesis of MIPs and nanoMIPs, with immense applications in the analysis of pharmaceutical compounds present in residual water and food matrices. Additionally, they have demonstrated potential as drug carriers in the field of cancer therapeutics and theragnostics [38,39,40,41,42]. This methodology is characterized by using only one functional monomer, typically methacrylic acid (MAA), and ethylene glycol dimethyl acrylate (EGDMA) as a cross-linker, which generates spherical particles with sizes ranging from 10 µm to 700 nm.

Ma Jing et al. reported a precipitation polymerization method combined with electrochemically mediated atom transfer for sialic acid printing, using 4-vinylphenylboronic acid as the functional monomer, obtaining nanoMIPs with sizes between 100 and 330 nm and narrow dispersion. This technique does not use a radical initiator but a catalytic agent [Cu II ((tris(2-pyridylmethyl)amine) Br] [Br] and ethyl 2-bromoisobutyrate as an initiator, which form alkyl halides and transition metal complexes. The carbon-halogen bond of the initiator is homolytically activated by the transition metal of the catalyst, producing the radical species to initiate polymerization. As activation only occurs in alkyl halide bonds, this makes this type of polymerization more controlled [43].

#### 2.1.3. Emulsion Polymerization

In emulsion polymerization, a surfactant is added to the polymerization mixture to stabilize the micelles produced between the monomer and the template molecule. On the other hand, the initiator is soluble in an aqueous medium and is not mixed directly with the monomer [44,45,46]. The surfactant promotes emulsification and solubilizes the monomer droplets within the micelles, allowing control over the number of nucleated particles. Additionally, the surfactant stabilizes the particles and produces more homogeneous particles. This technique yields spherical MIPs and nanometer-sized polymers known as nanoMIPs. In 2002, Vaihingen et al. [47] synthesized nanoMIPs for the first time using the miniemulsion polymerization technique for a chiral molecule, obtaining spherical nanoparticles with high affinity toward one of the enantiomers. The miniemulsion technique has been used in the synthesis of nanoMIPs for the removal of contaminants and medicines in water, as well as in the extraction of toxic substances in the food industry [48,49,50,51]. Ou et al. used the Pickering emulsion polymerization technique, which incorporates solid colloidal substances that act as stabilizers in immiscible solutions to form the emulsion [52]. They synthesized nanoMIPs using Fe_3_O_4_ as colloidal magnetic nanoparticles and chitosan as a stabilizer to print erythromycin [53], using MAA and EGDMA as the monomer and cross-linker, respectively. The nanoMIPs obtained had high specificity and selectivity against erythromycin, being reusable in several cycles of antibiotic removal. Other researchers have integrated various polymerization methods to produce novel molecularly imprinted polymers such as surface imprinting, core-shell, and solid-phase techniques.

#### 2.1.4. Surface Printing

The process of surface printing involves the deposition of the template molecule onto a substrate, which serves as a support for the subsequent analysis of the printed molecule. The substrate is activated on the surface with a molecule that has a vinyl group (carbon-carbon double bond) to enable surface printing, by adding the monomers and template molecule together with the initiator, generating micrometric-sized films (Figure 1F). This type of printing has been used in electrochemical sensors for the psychotropic drug chlorpromazine [54], in the extraction of bisphenol A from water remnants using graphene oxide as a support [55], and in the detection of cancer biomarkers using microfluidic paper as a substrate, where Qi et al. [56] used carcinoembryonic antigen as a template molecule. Surface printing was inspired by the adhesive of mollusks. Liang et al. [57] used a polyanionic membrane as support, dopamine hydrochloride (PDA) as the monomer, which polymerizes in alkaline media, and trypsin as a template molecule for the detection of bioanalytes. The polyanionic membranes were submerged in a solution of trypsin and PDA at pH 8.5 to carry out polymerization for 12 h. This study demonstrated that biological analytes, such as proteins and cells, can be detected rapidly, sensitively, and selectively without chemical labeling by using low-cost polymeric membrane ion-sensitive electrodes, which have been widely used in clinical analysis. Liang’s proposed biomimetic detection platform may have the potential to quantify many other targets, such as DNA, viruses, or even tissues, using MIPs.

#### 2.1.5. Core-Shell Molecular Printing

One of the approaches employed in molecular printing is the core-shell approach. However, it is important to note that this technique diverges in terms of substrate characteristics, namely, form and size. In the core-shell method, spherical substrates with nanometer dimensions are generally used to produce printed particles with sizes between nanometers and microns. The subsequent section provides a detailed description of these substrates.

Core-shell molecular printing is characterized as a heterogeneous methodology where different phases are distinguished in the synthesis process. In this method, emulsion, precipitation, and surface printing techniques are combined to produce a printed polymer with a generally inorganic core [58]. The resulting particle has a core-shell structure with a different composition on the surface compared to the interior. To achieve this, the shell polymer is added using free radical or controlled polymerization techniques. The thickness and composition of the cover can be controlled by adjusting the reaction conditions, such as monomer concentration, reaction time, and temperature (Figure 1G) [59]. This particular synthesis method has been used in various studies pertaining to protein recognition and enrichment [60,61,62], the isolation of bacteria such as Escherichia coli OP50 [63], and for drug transport. Hamid et al. [64] synthesized a core-shell polymer using doxorubicin as a template and the epitope of the HER2 protein, which is a receptor for human epidermal growth factor overexpressed in ovarian cancers. They used dopamine as the monomer and silica nanoparticles as the core. These nanoparticles were used for the targeted delivery of doxorubicin for the treatment of ovarian cancer in mice. The resulting nanoparticles had a size of 80 nm with spherical morphology, and the encapsulated drug had a rapid release of 4 h, with a maximum release at 24 h. These nanoparticles, used for monitoring ovarian cancer in mice, showed a decrease in tumor growth, resulting in an increased lifespan for the mice.

Another study by Jalilzadeh et al. synthesized microspheres with a magnetic core imprinted with adenosine to be detected in blood plasma [65]. High levels of adenosine in the blood are related to certain neurological diseases such as epilepsy, Parkinson’s, and schizophrenia, as well as cardiovascular diseases [66,67]. For the synthesis of these microspheres, they used hydroxyethyl methacrylate (HEMA), polyvinyl butyral, acrylic acid as the monomer, and adenosine as the template. The magnetic nucleus used was Fe_3_O_4_ spheres of 5 μm in diameter. To determine the adsorption efficiency of the microspheres imprinted with adenosine, they were compared with non-imprinted microspheres, performing thermodynamic and kinetic studies, which determined a limit of detection (LOD) of 1.9 nM. This demonstrated that these microspheres are highly sensitive in the detection of adenosine in plasma.

Another type of methodology to obtain printed nanoparticles is solid-phase synthesis, which is described below.

#### 2.1.6. Solid-Phase Printing

**Figure 2:**
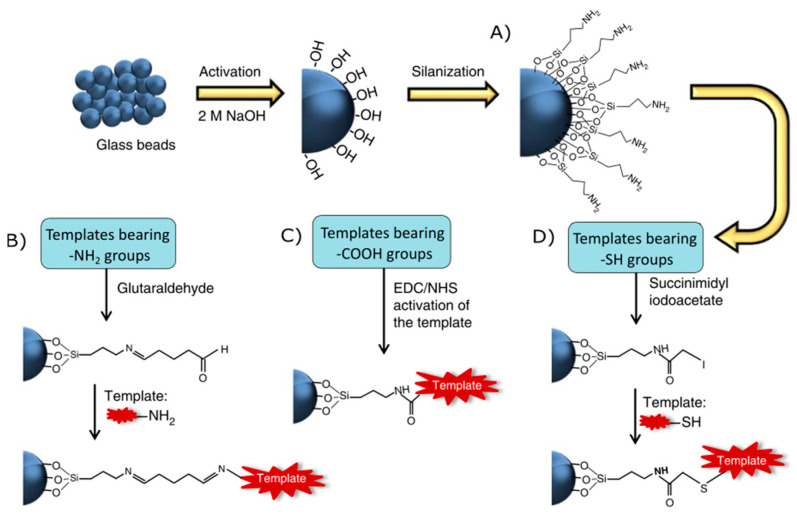
Solid-phase synthesis template molecule immobilization by different functional groups [68].

The solid-phase synthesis of nanoMIPs follows the same principles as molecularly imprinted polymers, with the difference that a solid phase is incorporated to immobilize the template molecule via covalent bonds in order to restrict the degrees of freedom of the molecule (Figure 1H) [68,69,70]. Canfarotta et al. [68] established three synthetic routes to immobilize the template molecules in the solid phase using three functional groups: amino (-NH_2_), carboxylic (-COOH), and thiol (-SH). The choice of the synthetic pathway is determined by the specific functional group present in the target molecule, as seen in Figure 2.

The initial step is the activation of the solid phase, typically comprising spheres of silicon oxide (SiO_2_). The reaction involves the utilization of sodium hydroxide (NaOH) to produce surface hydroxyl groups. Subsequently, a coupling agent containing silane groups and a terminal amino group, such as 3-aminopropyltriethoxysilane, is introduced. The immobilization of the template molecule occurs at the amino terminal (Figure 2A). For templates with an amino group, the solid phase must be activated with glutaraldehyde, generating terminal aldehyde groups that are reactive to the nucleophilic attack of the template (Figure 2B). To immobilize the template having a carboxylic group, the template must be activated using a carbodiimide cross-linker (EDC/NHS), producing a more reactive intermediate to anchor the template by the carboxylic group (Figure 2C). The final proposed route involves immobilizing the template molecule with a thiol group. In this case, the solid phase must be activated using a cross-linking agent for the thiol group, favoring the nucleophilic attack of the sulfide on the solid phase (Figure 2D). Once the template molecule is immobilized, the polymerization mixture is added, where the monomers are generally acrylate/methacrylate (methacrylic acid, ethylene glycol dimethacrylate, trimethylolpropane trimethacrylate, pentaerythritol tetrakis (3-mercaptopropionate), N-isopropylacrylamide, N-tert-butylacrylamide, acrylic acid) with the cross-linker being N,N′-methylenebisacrylamide. These components establish non-bonding, Van der Waals, and dipole-dipole interactions with the immobilized template molecule [71,72,73]. The polymerization is carried out with a radical initiator such as ammonium persulfate. For homolytic cleavage to occur and generate the radical at room temperature, a catalyst such as N,N,N′,N′-Tetramethylethylenediamine must be used [74]. By performing the polymerization at room temperature, the entropy of the non-binding interactions between the monomers and the immobilized template molecule is reduced, generating more specific cavities.

Finally, the nanoMIPs must be extracted from the solid phase. This is done by washing in a vacuum filtration system with deionized water at 0 °C to remove all the monomers and oligomers with low interaction with the template molecule, and then washing with water at 60 °C to break the non-binding interactions between the template molecule and the nanoMIPs, thus removing them from the solid phase [75]. The nanoMIPs obtained through this methodology have diameter ranges between 30 and 500 nm [70,76,77,78] and have been used in different types of sensors. This technique allows the printing of biomolecules because it can be polymerized at room temperature and in aqueous media, which makes it more suitable for biomolecules. Moczko et al. [79] developed an imprinted polymer for immunoglobulin G (IgG) using the complete biomolecule, a fragment (Fc domain), and an epitope as templates to determine if a synthetic equivalent of the protein can be produced. The IgG templates and Fc fragment were immobilized to the solid phase by amino groups, while the epitope was immobilized by the thiol group. The resulting nanoMIPs were analyzed with surface plasmon resonance (SPR) to determine the dissociation constants (Kd). NanoMIPs specific to the Fc domain had a Kd value of 7.6 nM, while those specific to the epitope had a Kd value of 2.5 nM.

The results obtained in this study affirm that it is possible to use the complete biomolecule as a template, such as the Fc domain, to obtain nanoMIPs with specificity towards immunoglobulin. However, they highlight that the use of the epitope is more economically advantageous, and the nanoMIPs produced with the epitope have nanomolar specificity, similar to those produced with other templates. Additionally, other studies have synthesized MIPs using biomolecules as templates and have achieved similar affinities to their natural counterparts. Bagán et al. [80] synthesized nanoMIPs for adult human hemoglobin (HbA) with a slight modification where the solid phase was dissolved in hydrofluoric acid (HF) to remove the nanoMIPs. The nanoMIPs were synthesized by employing HbA as a template, which was subsequently subjected to trypsin digestion to retain only the peptides bound to the solid phase. The resulting nanoMIPs were first analyzed by sodium dodecyl sulfate-polyacrylamide gel electrophoresis (SDS-PAGE) in a competitive assay with interfering proteins such as bovine serum albumin (BSA) and equine skeletal muscle myoglobin (Mb). Finally, these nanoMIPs were used for the selective separation of proteins, demonstrating that they act as specific templates for recombinant HbA and HbF, thus providing the possibility of purifying proteins and performing bioseparations on a large scale [80]. This type of solid-phase printing differs from Canfarotta since the resulting polymers are micrometric in size.

Molecularly imprinted polymers present different strategies to obtain a mold of controlled shape; some of these lack this control, such as bulk polymerization and precipitation where random conditions of temperature, agitation, and the difficulty of extraction of the template molecule provide irregular cavities. On the other hand, the most advanced techniques, such as surface printing, solid-phase printing, and core-shell, are polymers with more controlled cavities, obtained under less random synthesis conditions, with the desired applications. These latter synthesis methods are the most used in health area applications, partly due to the possibility of printing biomolecules by applying more stable methodologies for biomolecules with ambient temperature and physiological pH.

In recent years, there has been a significant expansion in the field of nanoMIPs, with numerous studies exploring diverse methodologies and templates to address a wide range of applications [81,82,83,84,85]. The growth they have exhibited is due to improvements in their surface properties compared to their MIP counterparts, as their nanoscopic size increases the contact surface and bonding kinetics [86]. Furthermore, the ability to fabricate biomolecules through printing techniques significantly expands the scope of scientific investigation. This enables the integration of biomolecules into diverse sensor platforms, thereby revolutionizing the fields of medicine and oncology. In the subsequent section, we shall explore the diverse methodologies employed in the utilization of MIPs for detecting a wide range of cancers.

## 3. Molecular Imprinting Polymers Used in Sensors for Cancers

### 3.1. Detection of Lung Cancer

Liquid chromatography coupled with mass spectrometry (LC-MS) is one of the techniques used to detect proteins, especially biomarkers. Due to the inherent sensitivity of this technique and the typically low abundance of target proteins in biological matrices, it is necessary to employ a pre-treatment step to concentrate the proteins of interest. Solid-phase extraction (SPE) represents one of the viable approaches to enhance protein concentration during analysis. A study conducted by Rossetti et al. [87] synthesized MIPs using the precipitation method to be used as a selective absorbent in the diagnosis and quantification of ProGastrin Releasing Peptide (ProGRP), consisting of an automated extraction based on MIPs and coupled to liquid chromatography (LC-MS). ProGRP is a sensitive and specific biomarker for small-cell lung cancer found in picomolar ranges in serum [88]. The template used was the peptide Z-NLLGLIEA (Nle), which was protected and modified to increase solubility in the polymerization solvent. The N-terminal was protected with benzyloxycarbonyl (Cbz, Z), and the C-terminal lysine was replaced by norleucine (Nle). The microspheres obtained were packed in a trap column for direct integration with LC-MS. Samples from patients with lung cancer were analyzed, thus making progress in initiating the first automated method for direct extraction of ProGRP with low limits and without the use of antibodies.

**Figure 3:**
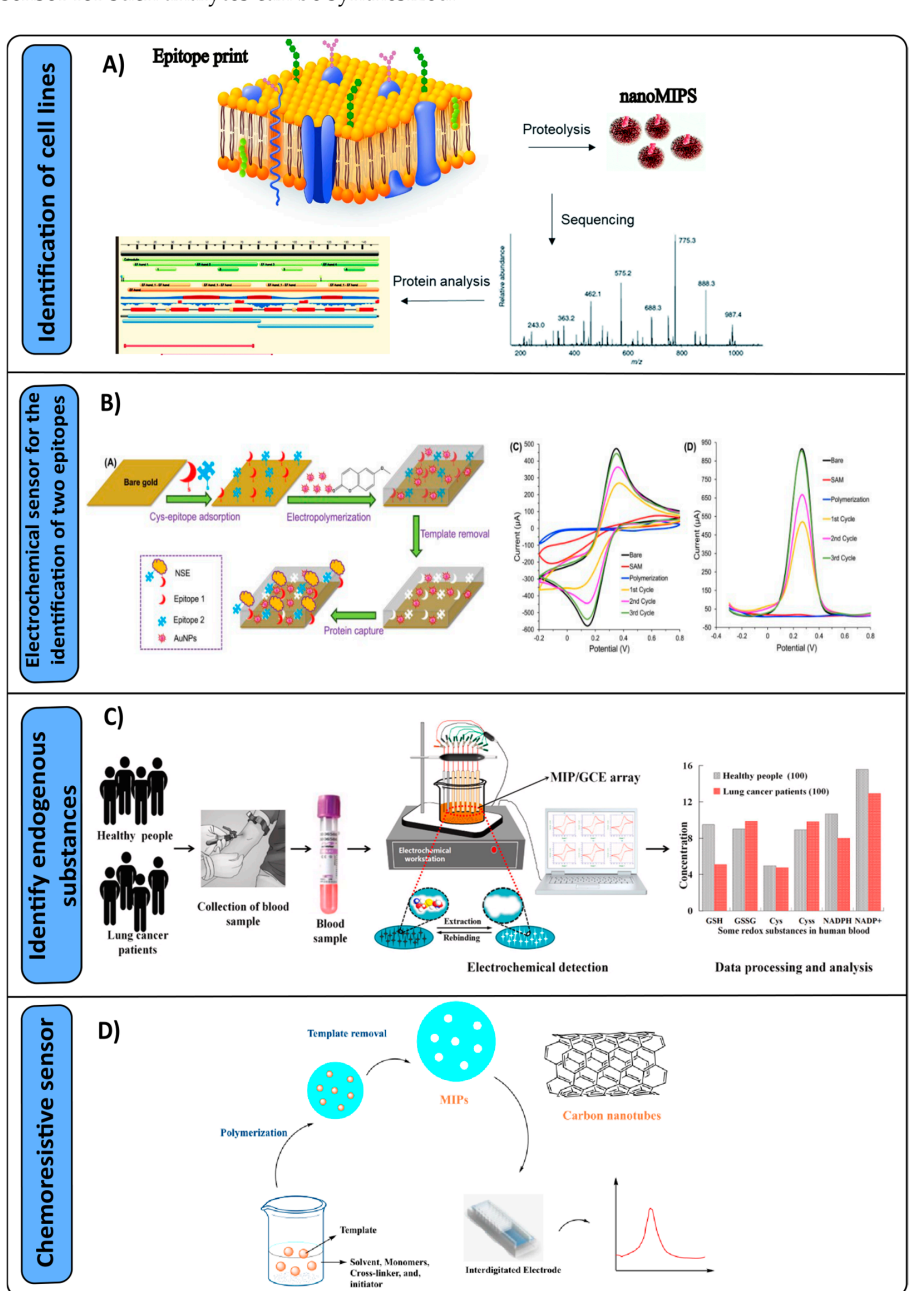
Sensors made with molecular imprinting polymers for the detection of lung cancer. (**A**) Proteomic analysis [90]. (**B**) Electrochemical sensor for epitope identification [91]. (**C**) Electrochemical sensor for the identification of endogenous substances [92]. (**D**) Chemoresistive sensor for the determination of hexanal [93].

A study by McKitterick et al. [89] also analyzed ProGRP with MIPs and LC-MS, but identified another low-abundance cancer cell biomarker known as neuron-specific enolase (NSE). They determined that the imprinted polymers had a selective affinity toward their respective peptide targets, managing to determine both biomarkers in nanomolar ranges with clinically relevant values. Another study conducted by Piletska et al. [90] also used LC-MS for the identification of lung cancer cell lines resistant to radiotherapy (Figure 3A). Four lines, A549, H460, H23, and H52, were studied as targets for MRC-5 normal lung fibroblasts. The proposed method was successful in comparing the proteomics of the surface of cancerous and normal cells and identifying biomarkers based on cellular resistance. They determined that the neutral amino acid transporter B(0) and the cell surface antigen chain 4F2 are present in greater amounts in radiation-resistant cells.

An alternative for the identification of biomarkers is electrochemical sensors, which use an electrode as a transducer element in the presence of an analyte. Pirzada et al. [91] developed an electrochemical sensor for the identification of two epitopes of NSE in human serum through surface polymerization obtained by electropolymerization, which were decorated with gold nanoparticles (Figure 3B). It was observed that the combination of two epitopes in the imprint led to a notable enhancement of 2.5 times in its sensitivity to NSE compared to the utilization of a single epitope. By integrating gold nanoparticles into the polymeric matrix, a significant enhancement in yield was observed. This novel approach enabled the detection of the NSE biomarker with remarkable sensitivity and specificity, with an impressive limit of detection (LOD) of 25 pg/mL. Liu et al. [92] developed an electrochemical sensor to identify six endogenous substances, namely, glutathione disulfide (GSSG), glutathione (GSH), cysteine (Cys), cystine (Cyss), β-nicotinamide adenine dinucleotide phosphate (NADP+), and reduced tetrasodium salt coenzyme II (NADPH). The polymerization was carried out in situ by electropolymerization using the same analytes as templates. The sensor was analyzed with blood samples from mice with lung cancer, obtaining detection values for the six analytes in the ranges of 10^−11^ to 10^−8^ M (Figure 3C) and an LOD of up to 20 pM, demonstrating that a highly sensitive and selective sensor for such analytes can be synthesized.

Gaseous tumor markers have emerged as significant contributors in the field of lung cancer research, with particular emphasis on hexanal. Janfaza et al. [93] developed a chemoresistive sensor for the identification of hexanal at room temperature using nanoMIPs and carbon nanotubes (Figure 3D). The response of the sensor increased proportionally to the hexanal concentration, with a detection limit of 10 ppm. Another study conducted by Chen et al. [94] developed a quartz crystal microbalance (QCM) gas sensor to detect hexanal, using a hydrophobic molecular imprinting polymer to prevent water vapor absorption during measurements. This sensor was tested with different gases as interferents, which did not have a significant response, while hexanal was detected sensitively and reliably in the range of 1 to 80 ppm. These results were supported by gas chromatography coupled with mass spectrometry, ensuring the sensitivity of the sensor. Volatile organic markers are an alternative to identify or monitor patients with possible lung cancer; another type of substance analyzed is nonanal. Jahangiri Manesh et al. [95] synthesized a chemoresistive sensor that contained gold nanoparticles and MIPs obtained by precipitation polymerization for the identification of nonanal. The sensor was selective, with an LOD of 4.5 ppm and a working range of 2.5 to 100 ppm. It is important to acknowledge that the development of sensors for the detection of volatile organic compounds as potential tumor markers represents a significant advancement in cancer diagnosis. These sensors contribute to the expanding number of existing markers and offer the potential to detect cancer at earlier stages. However, it is crucial to address the challenges associated with sensor sensitivity and the prevention of water condensation, as these factors can significantly impact the efficacy of the detection process.

It should be noted that the lung cancer markers mentioned in this review were obtained through surface printing techniques mediated by electropolymerization and others by the precipitation method. These techniques obtained micrometer-sized polymers ideal for applications in LC-MS and electrochemical sensors.

### 3.2. Breast Cancer Detection

In the realm of breast cancer detection, a diverse range of protein-based tumor markers has been extensively explored. However, it is worth noting that MIPs have also been investigated for their potential in this area. One of the proteins used as a tumor marker is transferrin (TfR), which can be overexpressed and is closely related to various cancers, including breast cancer [96]. This protein is responsible for transporting iron in blood plasma, and its soluble sTfR form can be detectable in blood serum. Proteomics has emerged as a prominent approach for the identification of compounds within complex biological systems. This methodology has gained significant importance in recent years, primarily owing to its capacity to accurately and sensitively quantify proteins. The current complexity of this approach lies in the preconcentration of proteins, as previously discussed in this literature review. This step typically involves the use of antibodies, which are unstable and costly, thereby restricting their widespread utilization. MIPs have emerged as a promising alternative to antibody-based recognition systems. This has sparked significant interest within the scientific community, leading to a surge in research efforts focused on exploring the potential of MIPs in conjunction with liquid chromatography and mass spectrometry techniques.

A study conducted by Liu et al. [97] synthesized MIPs using three peptides of different lengths as templates. The resulting MIPs were combined with tandem LC-MS-based targeted proteomics for sTfR measurement, and the MIPs were obtained by the precipitation method. They optimized the analysis method using MIPs and analyzed human serum samples. The results obtained showed a 12-fold improvement in sensitivity using MIPs, with a limit of quantification (LOQ) of 200 ng/mL, demonstrating the sensitivity of this method [98]. Another targeted proteomics study combining LC-MS/MS was conducted by Zhang et al. [99], where they used MIPs in the core-shell method for histone peptide enrichment. Post-translational modifications of histones, such as specific lysine residues, can be modified with one, two, or three methyl groups (mono-, di-, or tri-) or with an acetyl group, and these specific modifications are involved in various cancers, including breast cancer. In the study, they analyzed three types of peptides, some methylated and others acetylated, with three histone lysine sites: H3K79, H3K122, and H4K3 in breast cancer cell lines. They used a truncated peptide as a template molecule, which is shared by all the other histone peptides, thus achieving a 10-fold improvement in sensitivity using MIPs. This study marks the beginning of genome analysis using MIPs as recognition material in cancer cell lines. Other methodologies used for the detection of tumor markers include electrochemical sensors employing MIPs for the detection of carbohydrate antigen 15-3, DNA, and receptor 2 of the human epidermal growth factor (HER2-ECD) [98,100,101]. A study conducted by Lahcen et al. [102] used laser-engraved graphene (LSG) modified with gold nanostructures and MIPs by the surface imprinting method to detect HER2. The overexpression of this particular protein has been observed to be associated with the development of an aggressive subtype of breast cancer. Therefore, the timely detection of this protein is of utmost importance. Furthermore, the use of LSG has become attractive due to its efficiency, low cost, and increased sensitivity due to its active surface area. Lahcen [102] studied the first MIP-based LSG biosensor modified with gold nanostructures (AuNS) for the detection of HER2 in serum samples, focusing on a point-of-care (POC) device. This biosensor successfully detected HER2 in the concentration range of 1 to 200 ng/mL with a limit of detection (LOD) of 0.43 ng/mL.

In addition to conventional methods, alternative approaches for cancer detection involve fluorescence-based techniques, such as focal microscopy and immunoassays. Fluorescence stands out as a highly sensitive technique that identifies trace levels of analytes, making it one of the most sensitive methods available. MIPs have been strategically designed for the identification of antibodies in cancer cell lines, as well as for C-peptide in human urine [103]. Core-shell-type MIPs incorporating quantum dots have been synthesized and utilized to identify lysozyme, a protein associated with the carcinogenesis process. The developed MIPs demonstrated a limit of detection (LOD) of 3.2 μg/mL, indicating their sensitivity in detecting lysozyme. Furthermore, these MIPs have been effectively employed in the detection of lysozyme in human serum, highlighting their potential for clinical applications [104]. El-Schich et al. [105] developed a MIP for the detection of sialic acid applied in flow cytometry and focal microscopy. The polymer was synthesized by the core-shell methodology using sialic acid as a template and equipped with nitrobenzoxadiazole fluorescent indicator groups [106]. They demonstrated that the MIP recognizes breast cancer cells from epithelial cells (EpCAM expression), indicating that MIPs can be used as an additional tool to detect EpCAM-positive breast cancer cells.

The MIPs used in breast cancer detection mentioned in these studies obtained low detection limits in different techniques, such as LC-MS-based targeted proteomics and electrochemical sensors that analyzed blood serum samples. The polymers applied in these techniques had micrometric sizes. It should be noted that the study using the core-shell printing method with nanometric sizes used fluorescence techniques to identify antibodies in cancer cell lines and other biomarkers, demonstrating its potential for clinical applications due to its high sensitivity and low detection limits.

### 3.3. Detection of Cancer of the Digestive System

MIPs have also been implemented for the identification of tumor markers in the digestive system. Taheri et al. [107] implemented a dual detection approach for AFP and CEA, using nanostructured materials that have the potential to improve detection sensitivity in MIP sensors by incorporating two templates [108]. A conductive monomer, polypyrrole (Py), was used in conjunction with the morphology and charge transfer modifier methyl orange (MO) to obtain MIPs of dual-templated rectangular nanotubes on a fluorine-doped tin oxide electrode (FTO). The electropolymerization (surface polymerization) process produced a detection layer of FTO/PPy-MO DMIP that exhibited stability and high conductivity. This layer was used effectively for the detection of target analytes through their rebinding into the specific printed wells. Label-free sequential analysis of two or more analytes can be achieved using a one-for-one approach, where a single detection element is employed. Using this approach, the CEA-specific cavities were presaturated until no further increase in resistance to charge transfer (Rct) was observed. The cavities were then used for the measurement of alpha-fetoprotein (AFP), and the reverse strategy was employed to measure CEA. The DMIP sensor demonstrated detection limits of 1.6 and 3.3 pg/mL, indicating a high level of sensitivity and reproducibility. Additionally, the ease of fabrication makes it a suitable candidate for point-of-care applications. Glycoproteins are characterized by two distinct structural features, namely, the epitope and glycan. Zhou et al. [109] used the orthogonal dual molecularly imprinted polymer-based plasmonic immune sandwich assay (od-MIP-PISA) for the detection of CEA, commonly used as a marker for colon cancer. The methodology involves the use of two different types of molecularly imprinted polymers (MIPs) to achieve dual recognition of a target glycoprotein. This is achieved by using a slide coated with epitope-imprinted gold nanoparticles (AuNPs) as a capture substrate to recognize the peptide epitope and Raman-active silver nanoparticles imprinted with glycans as nanotags to recognize the glycans. This method facilitated the detection of CEA concentration in serum samples, allowing discrimination between colon cancer patients and healthy individuals. Speed, stability, fewer sample requirements, and cost-effectiveness were benefits of this approach. The printing techniques used exhibit wide applicability, allowing their extension to a wide range of glycoproteins, in addition to promoting the use of MIPs in the surface-enhanced Raman scattering (SERS) technique, which is known for being highly sensitive and non-destructive.

On the other hand, Piloto et al. [110] investigated the integration of cellulose-based hydrogel, quantum dots, and molecularly imprinted polymers in their study. The hydrogel functions as a support matrix, while the quantum dots serve as fluorescent markers for signal production and identification of the CA19-9 protein biomarker, which is linked to pancreatic cancer (PC). The proposed approach involves the use of MIPs as biorecognition elements, which are coupled with cadmium telluride (CdTe) quantum dots and semiconductor nanocrystals that serve as optical detection probes for the target analyte. The polymerization strategy employed is surface imprinting, which allows greater control over the growth of the polymer around the template molecule compared to traditional bulk polymerization methods. The HEC: MIP@QDs hydrogel sensor that was printed has demonstrated favorable characteristics such as a low detection limit, exceptional selectivity, reliable reproducibility, and stability, resulting in an optical diagnostic method capable of detecting CA19-9 in patients with pancreatic cancer. By integrating QDs into the MIP matrix, a simple approach to combining binding and reading functionalities is provided. This is achieved by the observable quenching of QDs upon interaction with target proteins. Lee et al. [111] synthesized a QDot composed of MIPs through the phase inversion process of poly(ethylene-co-vinyl alcohol) (EVAL) solutions. Solutions were prepared with different molar ratios of ethylene and included target molecules such as amylase, lipase, and lysozyme, which is one of the major protein components of saliva identified as a potential biomarker for pancreatic cancer. In the fabrication of MIPs for individual targets, spectrally unique quantum dots (QDs) were employed. Specifically, QD545 was used for lysozyme, QD605 for lipase, and QD655 for amylase.

The observed trend in the mean sizes of protein-printed MIP particles was found to be directly proportional to template concentration. The low cross-reactivity of composite particles made of QDs and MIPs indicates the potential for fluorescence-based detection of analytes by using distinctly colored QDs on separate MIPs, allowing the detection of template concentrations in real saliva samples. Selvam et al. [112] developed a ratiometric sensor capable of early detection of L-fucose (FU), a cancer marker commonly associated with hepatocellular carcinoma, pancreatic cancer, prostate cancer, colon cancer, and oral cancer. The present investigation focuses on the advancement and evaluation of a radiometric square wave voltammetric (SWV) sensor using the p-aminobenzoic acid (PABA)-FU molecularly imprinted polymer (MIP) combined platform. This sensor incorporated an amplification layer composed of Ag2Se@V2CTx to improve the characteristics of the signal. The composite material consisting of silver chalcogenide-loaded V2CTx MXene-MIP has demonstrated exceptional selectivity and sensitivity toward L-fucose, exhibiting a remarkable limit of detection (LOD) of 1.85 µM in PBS and 2.22 µM in blood serum. The characteristics of the robust ratiometric electrochemical sensor based on the relationship of the peak oxidation current demonstrated that it could be used in the detection of free FU in urine and serum FU (fucosylated Hpt) as a predictive marker for cancer, with low detection limits. The implementation of MIPs in the detection of tumor markers in the digestive system has proven to be an effective strategy to improve the sensitivity and specificity of cancer diagnosis. The developed techniques not only enable early and accurate detection of various biomarkers, but also offer cost-effective and adaptable methods for continuous patient monitoring.

### 3.4. Detection of Cancer of the Reproductive System

This section details sensors based on molecularly imprinted polymers (MIPs) for early detection of tumor markers related to male and female reproductive system cancers. Tumor markers related to cancers affecting the female and male reproductive systems have been successfully identified, and a subset of these markers has been incorporated into routine diagnostic tests. However, ongoing research is focused on investigating additional potential tumor markers, with the primary objective being the early detection of cancer. This early detection would facilitate intervention before the disease reaches an advanced stage. Here, we detail some of these studies using MIPs for the recognition of tumor markers.

A study conducted by Viswanathan et al. [113] developed molecularly imprinted gold nanoelectrodes (MIPGNEE) for the determination of CA 125. The sensor was fabricated by polyphenol electropolymerization using cyclic voltammetry. The template was incubated for 15 min at 4 °C at pH 7.4, followed by the application of voltage to re-cure. The developed sensor showed good increases in the studied concentration range from 0.5 to 400 U/mL, with a lower detection limit of 0.5 U/mL. On the other hand, Rebelo et al. [114] developed a surface plasmon resonance sensor for the identification of CA-125 using MIPs on a gold electrode. The synthesis of the MIPs was carried out by electropolymerization of pyrrole using cyclic voltammetry, achieving an LOD of 0.01 U/mL and a detection range between 0.01 and 500 U/mL. When comparing both investigations using CA 125 as the target analyte and the surface polymerization method, Rebelo’s investigation had a lower LOD and a wider detection range than Viswanathan’s method. Other research relating to the male reproductive system, such as that of Nguy et al. [115], synthesized a new type of screen-printed carbon electrodes (SPCE) that were modified with in situ synthesized gold nanoparticles (AuNP) for the MIP-based, label-free detection of sarcosine, an amino acid that serves as a biomarker of prostate cancer. AuNP preparation involved electrodeposition on SPCE, using the cyclic voltammetry (CV) scanning method. Subsequently, ultrathin layers of 4-amino thiophenol (p-ATP) were polymerized to generate MIP membranes with a microscale morphology exhibiting high conductivity. The results showed that the detection capabilities of MIP membranes produced on electrodes made of AuNPs and SPCE outperform those of MIP layers produced on traditional screen-printed gold electrodes (SPAuE), regardless of concentration. The cross-reactivity of the sensors was also evaluated using alanine and lysine, two compounds with remarkably similar chemical composition and molecular structure to sarcosine. The sensor demonstrated remarkable reproducibility, temporal stability, and surprisingly low cross-selectivity to other proteins. Sheydaei et al. [116] developed a modified electrochemical sensor based on MIP to detect traces of sarcosine (SAR) in urine samples. The polymerization process involved the use of methacrylic acid (MAA) and ethylene glycol dimethacrylate (EGDMA), with the aim of synthesizing a MIP capable of selectively binding to SAR. After successful elution of SAR from the MIP, the resulting leached MIP was used as a specific modifier on a carbon paste electrode (CPE). This modified CPE was then used for the in situ determination of SAR. The incorporation of MIP nanostructures in the paste composition produced a favorable electrocatalytic impact on the peak current. The sensor was evaluated to determine the SAR content in urine samples from both healthy individuals and cancer patients. The results indicated that the sensor demonstrated sensitivity at low SAR levels, suggesting its potential as a laboratory-scale diagnostic tool for the early detection of prostate cancer. The sensor demonstrates several advantageous features, including affordability, simplicity, favorable chemical stability, high repeatability, and accuracy, with a limit of detection (LOD) of 0.38 µM. Another investigation carried out by Puig et al. [117] successfully fabricated a nanocomposite sensor using a molecularly imprinted silicon polymer (MIP-Si) material. This all-solid-state potentiometric (ASS) sensor was specifically designed for the detection and quantification of sarcosine. The use of silica nanoparticles in the manufacture of MIP is motivated by their ability to increase stability, permeability, thermostability, and biocompatibility. MIPs were synthesized using MAA as the functional monomer, acetonitrile as the solvent, EGDMA as the cross-linker, and 2,2′-azobis(isobutyronitrile) (AIBN) as the radical initiator. The sensor manufacturing process involved the use of a mixture of conductive materials, which was subsequently injected into micropipette tips. In addition, the tip surface was coated with selective membranes incorporating MIPs to facilitate the potentiometric analysis of sarcosine. The sensor exhibited a remarkable degree of selectivity toward sarcosine when tested in simulated body fluid. It demonstrated a low detection limit of 7.8 nM and exhibited stability for a minimum of 150 days, yielding highly consistent and reproducible results, in addition to obtaining one of the lowest detection limits compared to other research in the detection of sarcosine. When comparing the research carried out for the detection of sarcosine using MIPs as recognition material, the research carried out by Puig has a lower LOD (higher sensitivity) compared to the other methods. Nguy’s method stands out for its high conductivity and low cross-selectivity.

**Figure 4:**
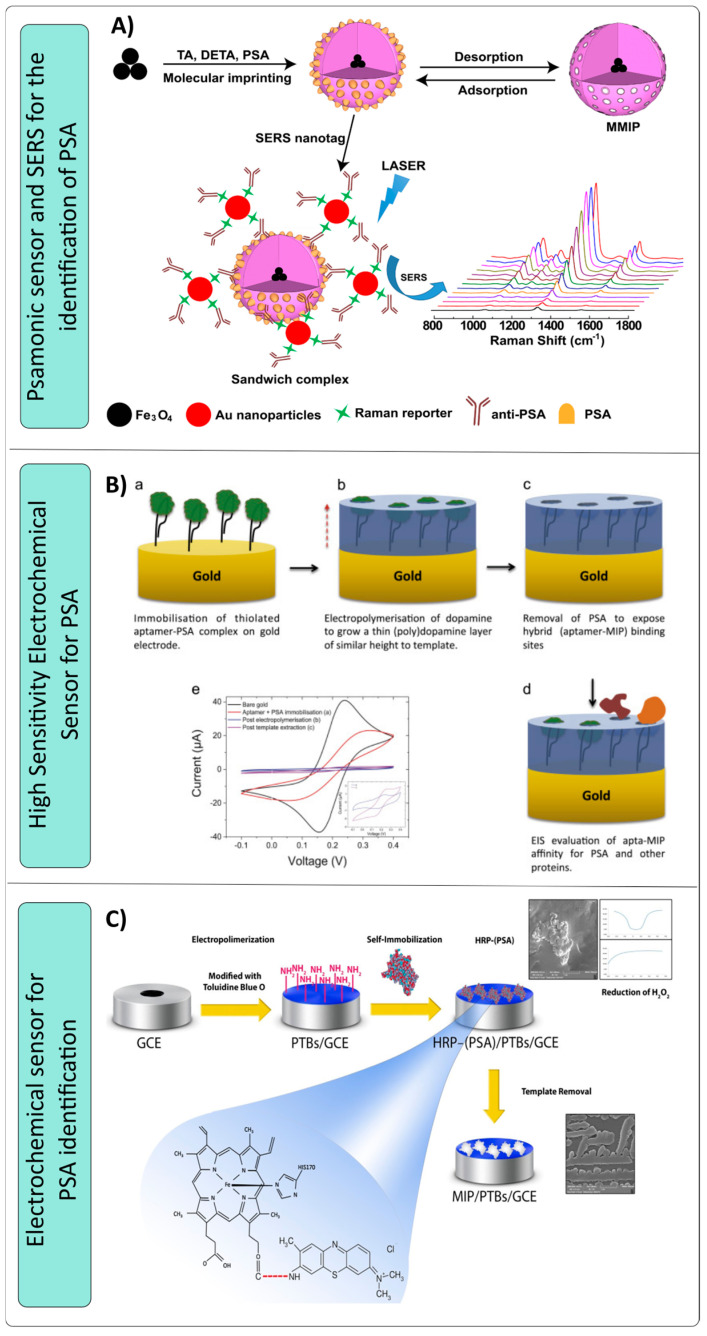
Schematics of sensors designed for the detection of tumor markers for the reproductive system using MIPs as identification material. (**A**) Sensitive and specific plasmonic biosensor for the detection of PSA [118]. (**B**) Electrochemical sensor aimed at the quantitative analysis of PSA [120]. (**C**) Electrochemical sensor for the identification of PSA [121].

By combining molecularly magnetic imprinted polymer (MMIP) and surface-enhanced Raman spectroscopy, Turan et al. [118] designed a sensitive and specific plasmonic biosensor for the detection of prostate-specific antigen (PSA) (Figure 4A). Tannic acid was used as the functional monomer, diethylenetriamine as the cross-linker, the tumor marker PSA served as the template molecule, and the polymerization method was the core-shell. The MMIP nanoparticles were conjugated with gold nanoparticles functionalized with anti-PSA and a Raman reporter, specifically 5,5′-dithiobis-(2-nitrobenzoic acid) (anti-PSA@DTNB@Au). Tannic acid, as a new functional monomer, has abundant galloyl groups that contribute to its favorable solid-liquid interfacial characteristics. The determined sensors LOD and LOQ were found to be 0.9 pg/mL and 3.2 pg/mL, respectively. The proposed methodology has exhibited numerous advantages, including its cost-effectiveness, reduced detection process, rapid response, increased sensitivity, and selectivity, as well as its suitability for quantifying PSA in serum samples. Matsumoto et al. [119] synthesized a surface plasmon resonance (SPR) sensor with 4-[2-(N-methacrylamido)ethylaminomethyl] benzoic acid (FM), 2-methacryloyloxyethyl phosphorylcholine (MPC), and N,N’-methylenebisacrylamide (MBAA) as functional monomer, comonomer, and cross-linker, respectively, for radical copolymerization to obtain PSA MIP. They implemented a post-printing strategy by treating with a capping agent that selectively inactivates low-affinity recognition cavities while capping high-affinity cavities with the addition of a low concentration of PSA as a dynamic capping agent. The nanocavities possess boronic acid groups and carboxy groups as sites to interact with carbohydrates and polypeptides, respectively. Treatment with a novel PEG-based capping agent selectively inactivated the low-affinity recognition cavities in the MIP thin layer while dynamically capping the high-affinity recognition cavities, resulting in high-affinity PSA-imprinted nanocavities for the detection of prostate cancer. The proposed strategy, which is a non-covalent molecular imprinting approach complemented by a PIM-based protection treatment, presents a pioneering methodology for the fabrication of glycoprotein recognition materials with exceptional sensitivity and selectivity, with a detection limit of 5.4 ng/mL.

Jolly et al. [120] synthesized a hybrid MIP electrochemical sensor aimed at the quantitative analysis of prostate-specific antigen (PSA) (Figure 4B). The surface of a pristine gold electrode was used to immobilize a preformed complex consisting of a thiolated DNA aptamer and prostate-specific isolate (PSA). The favorability of the order and homogeneity of the hybrid system was improved by immobilizing the complex on the surface prior to polymerization. Next, several layers of electropolymerized polydopamine were applied as a scaffold to provide support and protection to the aptamer. Furthermore, these layers were used to confine the aptamer within or near its preferred binding conformation. After the removal of the PSA (template), the relinking process between the restricted aptamer and the binding pocket of the polymer would occur, leading to the formation of a molded hybrid surface, known as apta-MIP, which possesses improved PSA binding properties compared to the aptamer alone. Electrochemical impedance spectroscopy (EIS) was used to evaluate the binding characteristics of the apta-MIP sensor for PSA, with an LOD of 1 pg/mL, obtaining a surprisingly low value for detecting this tumor marker with an LOD similar to conventional techniques such as ELISA

Sardaremelli et al. [121] developed a bioprinted polymer using a simple, effective, and inexpensive approach. This involved the electropolymerization of toluidine blue and autoimmobilization of horseradish peroxidase (HRP)-conjugated prostate-specific antigen on a glass carbon electrode (Figure 4C). Polytoluidine blue (P(TB)) possesses a substantial surface area that facilitates efficient loading of the HRP-PSA antibody onto the surface of a glassy carbon electrode (GCE). This process improves the efficiency of molecular printing, as well as the conductivity and electron transfer capabilities of the electrode. Hydrogen peroxide (H_2_O_2_) concentration was determined using three electrochemical techniques: differential pulse voltammetry, square wave voltammetry, and chronoamperometry. The bioprinted polymer was successfully used to quantify H_2_O_2_ in raw human plasma samples, achieving a low limit of quantification (LOQ) of 0.001 mM. This quantification limit is within the values for diagnosing prostate cancer, but the work reported by Jolly [119] separates it in sensitivity. When comparing all these sensors that used MIPs for the diagnosis of PSA, the method of Turan et al. has the lowest LOD (0.9 pg/mL), closely followed by the method of Jolly et al. (1 pg/mL). The method of Matsumoto et al. has a significantly higher LOD. The method of Sardaremelli et al. stands out for its applicability in human plasma samples

Electrochemical sensors have shown significant potential in detecting biomarkers for various types of cancer. This technology, which includes the use of molecularly imprinted polymers (MIPs) and nanoparticles, offers crucial advantages such as high sensitivity, selectivity, and low limits of detection (LOD). Integrating molecularly imprinted polymers (MIPs) with these recognition sensors further enhances their potential by enabling the detection of complex biomarkers, including high molecular weight molecules, bacteria, and viruses. Advanced techniques such as electro-polymerization and microfluidics are essential for developing these biosensors, as they facilitate the seamless integration of various analytical steps.

## 4. Conclusions

The field of tumor markers is currently in a state of continuous development. To date, no marker has been identified that can effectively detect cancer in its early stages. Furthermore, the identification of a single molecule is insufficient to conclusively determine the presence of cancer in a patient. It is worth noting that the timely detection of biomarkers in the early stages of cancer has been shown to significantly improve the prognosis and overall survival rate of patients. MIPs have not been overlooked in the realm of scientific research due to their notable attributes. These polymers exhibit exceptional versatility as they can be tailored to a wide range of analytes, including tumor markers. Furthermore, the possibility of replacing antibodies with MIPs offers important advantages, such as greater durability and adaptability. Different synthetic MIP techniques allow the printing of templates that can be used in various types of sensors, enabling their integration into different analytical devices for cancer diagnosis. The current state of printing biological molecules, including antibodies and proteins, remains in the development stage. This is mainly due to the inherent difficulty in ensuring the stability of these target molecules within the reaction environment, which is crucial for the formation of an optimal cavity. Actual challenges of molecularly imprinted polymer technology include the recognition of high molecular weight biomarkers in body fluids, or even bacteria and virus. The direct binding of a virus on the biomimetic site of MIPs (prepared by using the virus as template) could be monitored by stimulated emission depletion (STED) microscopy. The integration of these MIPs with transducers for fabrication of biosensors is also a research target. Electrochemical biosensors have attracted a lot of attention, due to their inherent properties, such as good sensititivity, easy miniaturization and low cost. The actual trends relay mainly on the use of electropolymerization of the monomers in the presence of the template and microfluidics to integrate different steps of the analysis.

Researchers involved in the studies mentioned in this review directed their efforts toward the application of biomarkers in various sensing technologies, such as surface plasmon resonance, electrochemical, chemoresistive, fluorescence, and ELISA sensors. Furthermore, they explored the use of biomarkers in the field of proteomics, successfully achieving impressive levels of sensitivity in detection. However, there is still much work to be conducted to effectively incorporate these materials into clinical diagnostic methodologies, particularly in the context of monitoring disease recurrence in patients. While it is important to note that the studies discussed here are currently in the development phase, they show promise as potential tools to facilitate the clinical diagnosis of various cancer types. Additionally, MIPs facilitate the selective recognition of biomarkers, considering the complexity of identifying some biomolecules due to the sample matrix or the tumor environment where they are present, as well as the large-scale manufacturing or reuse of the devices, which results in a decrease in implementation costs. As the use of MIPs for cancer diagnosis becomes increasingly prevalent, it is essential not to overlook considerations of their reusability, environmental impact, and the feasibility of sustainable production methods. This encompasses ensuring that these innovative sensors contribute to ecological sustainability in the long term.

In the future, it is expected that MIPs can be positioned as both selective and specific recognition materials for tumor markers, aiding in the prevention and diagnosis of cancer.
